# A retrospective analysis of the microbiology of diabetic foot infections at a Scottish tertiary hospital

**DOI:** 10.1186/s12879-020-4923-1

**Published:** 2020-03-12

**Authors:** Katherine E. Macdonald, Crispin Y. Jordan, Emma Crichton, Judith E. Barnes, Gillian E. Harkin, Lesley M. L. Hall, Joshua D. Jones

**Affiliations:** 1 Edinburgh Medical School: Biomedical Sciences, Infection Medicine, University of Edinburgh, Chancellor’s Building, 49 Little France Crescent, Edinburgh, EH16 4SB UK; 2 Biomedical Teaching Organisation, Edinburgh Medical School: Biomedical Sciences, University of Edinburgh, Doorway 3, Teviot Place, Edinburgh, EH8 9AG UK; 3 Diabetes and Endocrinology, Queen Elizabeth University Hospital, 1345 Govan Road, Govan, Glasgow, G51 4TF UK; 4 ZJU-UoE Institute, Zhejiang University School of Medicine, International Campus, Zhejiang University, 718 East Haizhou Road, Haining, Zhejiang, 314400 People’s Republic of China

**Keywords:** Diabetic foot infection, Microbiology, Retrospective, *Staphylococcus aureus*

## Abstract

**Background:**

This study represents the first Scottish retrospective analysis of the microbiology of diabetic foot infections (DFIs). The aims were to compare the microbiological profile of DFIs treated at a Scottish tertiary hospital to that in the literature, gather data regarding antimicrobial resistance and investigate potential trends between the microbiological results and nature or site of the clinical sample taken and age or gender of the patients.

**Methods:**

A retrospective analysis of wound microbiology results was performed, data were obtained from one multidisciplinary outpatient foot clinic during the 12 months of the year 2017. Seventy-three patients and 200 microbiological investigations were included. In cases of soft tissue infection, the deepest part of a cleansed and debrided wound was sampled. In cases of osteomyelitis a bone biopsy was obtained. Factors influencing the pattern of microbial growth or prevalence of *Staphylococcus aureus* were investigated.

**Results:**

Of the 200 microbiological investigations, 62% were culture positive, of which 37.9% were polymicrobial and 62.1% monomicrobial. Among the monomicrobial results (*n* = 77), most were Gram positive isolates (96.1%) and the most frequently isolated bacteria was *S. aureus* (84.4%). No methicillin-resistant *S. aureus* was reported. The prevalence of *S. aureus* in DFIs was associated with increasing age (*p* = 0.021), but no evidence of association with gender, anatomical sample site or sample material was found.

**Conclusion:**

The microbiological profile of DFIs in Scotland resembles that reported elsewhere in the UK. In this context, Gram positive organisms, primarily *S. aureus*, are most frequently isolated from DFIs. The *S. aureus* isolates identified were largely susceptible to antibiotic therapy. An association between increasing patient age and the prevalence of *S. aureus* in DFIs was observed.

## Background

Diabetes mellitus, herein diabetes, is the most common metabolic disease in the world. In 2014 there were an estimated 422 million adults living with diabetes, and the global prevalence continues to increase, with a projected 592 million people living with the condition by 2035 [1, 2]. Around one third of patients with diabetes will develop a diabetic foot ulcer (DFU) and approximately half of these will become infected [3]. DFUs develop secondary to a variety of factors, including peripheral neuropathy and peripheral arterial disease [4]. Diabetes in Scotland represents a significant burden, with nearly 300,000 people registered as having the condition in 2017 [5]. Many of these individuals (44.2%) have had diabetes for more than 20 years [5], a significant risk factor for the development of a DFU [6].

Infected DFUs are known as diabetic foot infections (DFIs) and carry a high burden morbidity and increased risk of mortality [3]. The microorganisms most commonly isolated from DFIs are aerobic Gram positive bacteria, predominantly *Staphylococci* [7, 8]. The prevalence of methicillin resistant *Staphylococcus aureus* (MRSA) in DFIs is considered to be 15–30% [8, 9]. Other bacterial genera commonly found in DFIs include *Streptococci, Enterococci, Enterobacteriaceae*, and *Pseudomonas* [3]. These infections are often polymicrobial and understanding the melange of species present can help inform clinical practice. Treatment for DFIs consists of wound care and antibiotics. The chronic and recalcitrant nature of these infections typically requires the administration of long courses of antibiotics, which may contribute to antimicrobial resistance. The efficacy of haematogenously distributed antibiotics may be further hampered by peripheral arterial disease [10].

An understanding of the microbiology of DFIs is important to inform clinical practice, offer insight into local microbiological trends and direct novel therapeutic strategies. To date, there has been just one small prospective study of the microbiology of DFIs in Scotland [11]. To supplement this, we therefore undertook the first Scottish retrospective analysis of the microbiology of DFIs. All DFI patients treated at an outpatient clinic at the Queen Elizabeth University Hospital (QEUH) in Glasgow during the 12 months of 2017 were included. The objectives were to compare the local microbiological profile to that in the literature, gather data regarding antimicrobial resistance and investigate potential relationships between the microbiological results and nature or site of the clinical sample taken and age or gender of the patients.

## Methods

This retrospective cohort study was undertaken in the multidisciplinary diabetic foot service at the tertiary Queen Elizabeth University Hospital in Glasgow, Scotland. The results of all microbiological investigations ordered by one multidisciplinary outpatient foot clinic during the 12 months of the year 2017 were retrieved by members of the direct care team. The age (years) and gender of each patient were recorded. Further information about the diabetes type of these patients could not readily be obtained due to the division of patient data between discrete electronic platforms. Microbiological investigations were undertaken, prior to the administration of antibiotics. Wound sampling was performed according to the National Institute for Health and Clinical Excellence guidelines for diabetic foot problems 1.6.1 [12]. All samples were taken from the base of a cleansed and debrided wound. In cases of soft tissue infection, a deep wound swab was taken from the debrided ulcer base. Bone biopsies were obtained in cases of suspected osteomyelitis. For all investigations, care was taken to prevent contamination with skin flora and provide clinically relevant results to inform patient care. All microbiological investigations were performed according to the UK Standards for Microbiology Investigations and local microbiological guidelines. Deep tissue swabs were placed on Amies transport medium with charcoal and sent to the Queen Elizabeth University Hospital Microbiology Department. Bone biopsies were placed in a sterile universal container. All samples were cultured for aerobic and anaerobic microorganisms, pure cultures were obtained and subjected to antibiotic sensitivity testing. The presence of coagulase negative *Staphylococci* in deep tissue swabs is routinely presumed to indocate skin flora and is not reported as significant growth. The following data were recorded for each result: investigation site (broadly divided into foot, toe or ankle), material sampled (bone biopsy or deep tissue swab) and general bacterial growth (no significant growth, monomicrobial or polymicrobial). For monomicrobial plates, the species identified were recorded, alongside any available antimicrobial sensitivity data. All data were anonymised before secure transmission from NHS Greater Glasgow & Clyde to the University of Edinburgh. This study was performed as a quality improvement study and Caldicott Guardian approved by NHS Greater Glasgow and Clyde.

Statistical analyses were performed using RStudio (version 1.1456) and Microsoft Excel. In R, the “lme4” and “lmerTest” packages were utilised [13, 14]. Four generalised linear mixed-effects model were run, taking into account the random effects of patients, to analyse the statistical significance of each parameter recorded in turn (the four independent variables: age, gender, investigation site and material sampled) in influencing the prevalence of *S. aureus*. The dependent variable in our models was the binomial response of whether an investigation signalled a *S. aureus* infection (‘yes’ vs. ‘no’); therefore, our models assumed a binomial distribution. We report our model results in terms of the ‘log odds’ that a given clinical sample is infected, log(*p*/(1-*p*)), where *p* equals the probability that a sample is infected. Therefore, *p*/(1-*p*) represents the odds of infection. For example, if the probability of being infected is 0.8, then the odds of being infected equals four (0.8/0.2). The ‘Diagnostics for HierArchical Regression Models’ (DHARMa) package was used to check the residuals for the mixed effects model [15]. Microsoft Excel was used to perform descriptive statistics on age, clinical microbiology and antimicrobial resistance.

## Results

### Study population

**Fig. 1:**
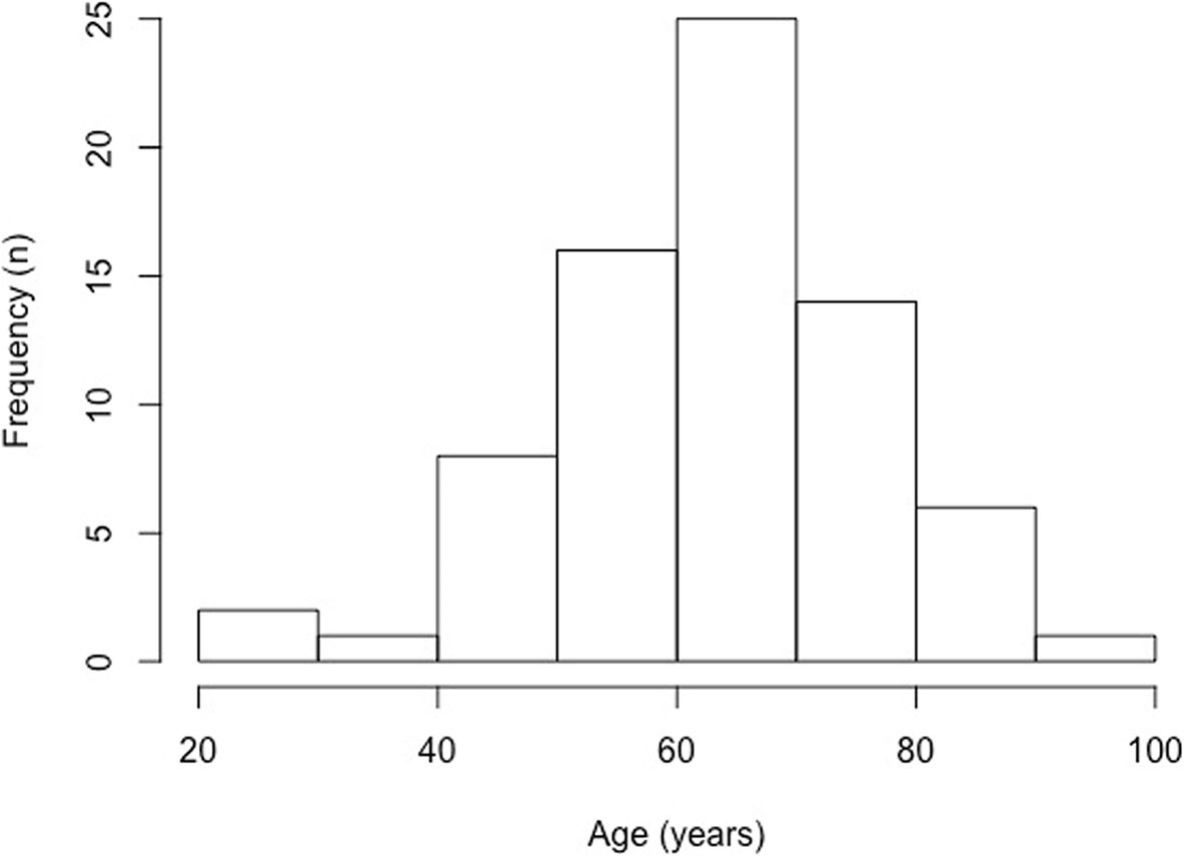
The age distribution of the diabetic foot patients included in this study (*n* = 73)

Seventy-three patients from one multi-disciplinary diabetic foot outpatient clinic had one or more microbiological investigations during 2017 and were therefore included in this retrospective study. In cases of soft tissue infection, deep tissue swabs were taken from the cleansed and debrided ulcer base. Bone biopsies were obtained in suspected cases of osteomyelitis. A total of 201 microbiological investigations were performed, however microbiological data for one monomicrobial deep tissue swab result were missing and this result was excluded; therefore, 200 microbiolocial results were included in this analysis. The study population comprised 56 males and 17 females. The age of the patients ranged from 28 to 94, with an average age (± standard deviation) of 63.8 ± 12.8 years (Fig. 1).

### The effect of gender, swab site and sample material on the nature of the swab result

Of the 200 microbiological investigations, 62% were culture positive, 37.9% of which were polymicrobial and 62.1% were monomicrobial. The distribution of the 200 results by gender, anatomical sample site and material sampled is shown in Table 1. About a third (38.1% male; 37.5% female) of investigations did not yield significant growth (i.e. these samples were sterile or represented presumed skin flora and were not reported by the Microbiology Department as significant). Among those infected, the majority had monomicrobial infections. A high proportion of monomicrobial results was also observed among clinical samples taken from foot (42.4%) or toe (36.2%) infections and from deep tissue (39.3%) swabs. Few clinical samples were obtained from the ankle (*n* = 3) and there were few bone biopsy (*n* = 9) results available. A Chi-Square test could not be performed with these data because of violation of the assumption of independence as some patients provided multiple sample results.

**Table 1:** The relationship between gender, sample site and sample type and the pattern of microbial growth observed

	Pattern of microbial growth			Total n (100%)
	Monomicrobial n (%)	Polymicrobial n (%)	No significant growth n (%)	
All clinical samples	77 (38.5%)	47 (23.5%)	76 (38.0%)	200
Male	62 (36.9%)	42 (25.0%)	64 (38.1%)	168
Female	15 (46.9%)	5 (15.6%)	12 (37.5%)	32
Ankle	0 (0.0%)	0 (0.0%)	3 (100%)	3
Foot	39 (42.4%)	22 (23.9%)	31 (33.7%)	92
Toe	38 (36.2%)	25 (23.8%)	42 (40.0%)	105
Bone biopsy	2 (22.2%)	6 (66.7%)	1 (11.1%)	9
Deep tissue swab	75 (39.3%)	41 (21.5%)	75 (39.3%)	191

### The microbiological profile of diabetic foot infection

The mean number of microbiological results per patient was 2.74 with a range of one to nine results and a mode of one. In some cases, the results of patients who had had multiple investigations were identical. However, clinical staff at QEUH advised that microbiological investigations are only routinely taken at the start of an infectious episode, with patients then being treated until clinical resolution of infection. Additional investigations in the same patient therefore likely reflect a new episode of infection. Therefore, all results were included in the analyses.

Among the results, 124 were positive for microbial growth, representing 1.70 positive cultures per patient. An exact mean number of isolates per patients could not be calculated given the polymicrobial nature of some results. The microorganisms isolated are detailed in Table 2.

**Table 2:** The microorganisms reported from diabetic foot wound cultures (*n* = 200)

		Number of microbiological results
Gram positive	*Staphylococcus aureus*	65
	*Staphylococcus epidermidis*	1
	*Staphylococcus lugdenensis*	2
	Group B *Streptococcus*	2
	Group C *Streptococcus*	1
	Group F *Streptococcus*	1
	Group G *Streptococcus*	1
	*Streptococcus constellatus*	1
	**Total Gram positive**	**74**
Gram negative	*Enterobacter hormaechei*	1
	*Escherichia coli*	1
	*Pseudomonas aeruginosa*	1
	**Total Gram negative**	**3**
	**Total monomicrobial swabs**	**77**
	Yeasts	1
	Mixed aerobic	40
	Mixed anaerobic	6
	Total positive swabs	124
	No significant growth	76
	**Total**	200

Excluding fungal, mixed and samples without significant growth, there were 77 monomicrobial results, of which 96.1% were Gram positive bacterial species and 3.9% were Gram negative. On a per patient basis (*n* = 73), only 4.1% of individuals ever grew a Gram negative species, and 43 patients tested positive on at least one occasion for a Gram positive bacterium.

### The prevalence of *S. aureus* in diabetic foot infections was influenced by age but not gender, anatomical sample site or sample material

**Fig. 2:**
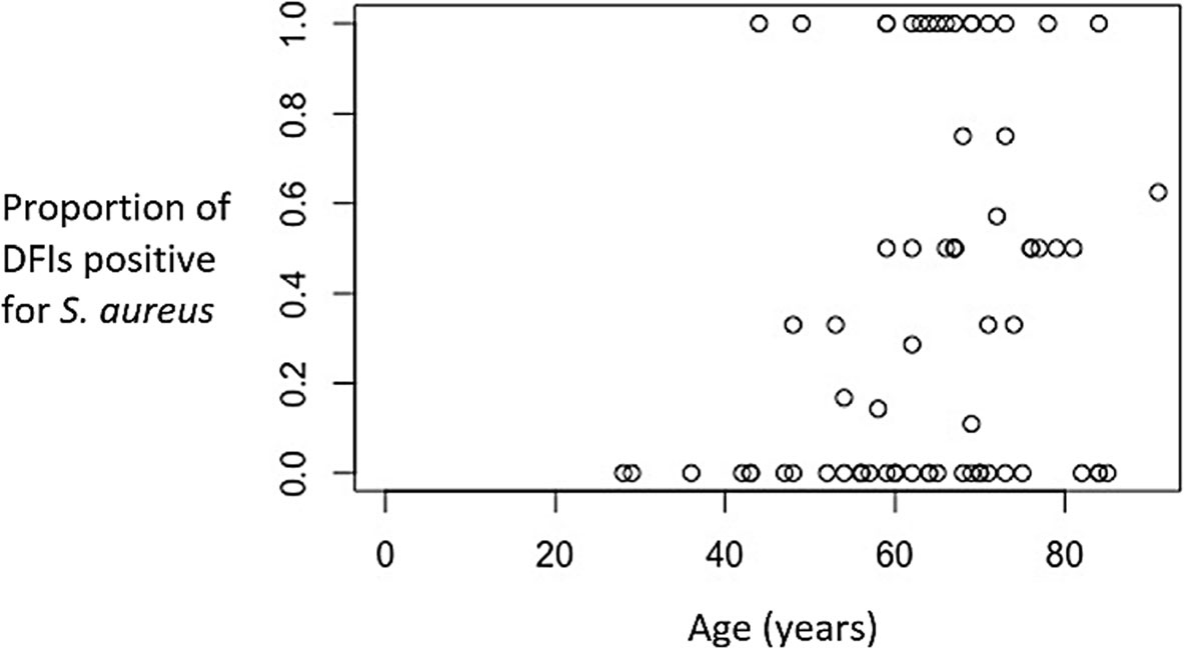
The effect of age on the prevalence of *S. aureus* among diabetic foot infections (*n* = 73)

*Staphylococcus aureus* was found in 32.5% of all results (*n* = 200) and accounted for 84.4% of the 77 monomicrobial results. We investigated whether the prevalence of *S. aureus* was related to gender, anatomical sample site or sample material using separate generalised linear models with mixed effects for each variable (Table 3). We found that the prevalence of *S. aureus was* not influenced by gender (*p* = 0.249), anatomical site (*p* = 0.202) or sample material (*p* = 0.556). A fourth generalised linear mixed-effects model indicated that the prevalence of *S. aureus* increased with age (*p* = 0.0211). The significance of this effect persisted following exclusion of the three youngest patients (potential outliers; aged 28, 29 and 36; *p* = 0.0451). We found that the mean change in log odds of being infected with *S. aureus* increases by 0.0372 ± 0.0161 annually (equivalently, each year, the odds of infection increase multiplicatively by exp.(0.0372)), with an intercept of − 3.24. For example, at age 40 the predicted probability (i.e., out of 1) of having a *S. aureus* positive DFI is 0.15. Once a patient reaches age 60, this increases to 0.27. Finally, by age 80 the predicted probability reaches 0.43. Thus, with increasing age the probability of having a *S. aureus* positive diabetic foot infection becomes more likely. Analyses with DHARMa indicated that our data met the assumptions of our model. To visualise this effect, we calculated the proportion of *S. aureus* positive results per patient, which ranged from 0 (if all swabs were negative) to 1 (if all results were positive), and plotted these data against the age for each individual (Fig. 2).

**Table 3:** The effect of gender, anatomical swab site and sample material on the prevalence of *S. aureus* among DFI patients

The prevalence of *S. aureus* in:			
	All results (*n* = 200)	Monomicrobial results (*n* = 77)	*P* value
	32.5% (*n* = 200)	84.4% (*n* = 65)	-
Male	30.3% (*n* = 168)	82.3% (*n* = 62)	-
Female	43.8% (*n* = 32)	93.33% (*n* = 15)	(*p* = 0.249)
Ankle	0% (*n* = 3)	0% (*n* = 3)	-
Foot	38% (*n* = 92)	78.9% (*n* = 38)	-
Toe	28.5% (*n* = 105)	89.7% (*n* = 39)	(*p* = 0.202)
Bone biospy	22.2% (*n* = 9)	100% (*n* = 2)	-
Deep tissue swab	33.3% (*n* = 191)	84% (*n* = 75)	(*p* = 0.556)

### Antimicrobial resistance of *S. aureus*

Finally, we examined the available antimicrobial sensitivity data. For some patients, antimicrobial resistance was investigated at multiple clinic visits. A total of 62 microbiological results from 38 *S. aureus* positive patients were tested for flucloxacillin resistance, with 95.2% of *S. aureus* isolates found to be sensitive. Clarithromycin sensitivity was investigated for 62 *S. aureus* isolates obtained from 38 patients, two of which were not tested for flucloxacillin resistance; of these 72.6% of *S. aureus* isolates were found to be sensitive. A small number of other *S. aureus* antibiotic sensitivity investigations were requested, with 95% sensitivity recorded against doxycycline (*n* = 20) and 100% sensitivity to penicillin (*n* = 8), clindamycin (*n* = 4) and vancomycin (*n* = 1). Notably, no MRSA was detected in this study.

## Discussion

This study represents the first retrospective analysis of the microbiology of DFIs undertaken in Scotland. The aim was to investigate whether the microbiological profile of diabetic foot infections at a Scottish hospital was comparable to previously published reports. The literature suggests that in the UK *Staphylococci*, particularly *S. aureus*, are most commonly isolated from DFIs [16–19], and the findings of this study support this. A previous prospective culture-based microbiological analysis of 20 Scottish DFI samples also identified *S. aureus* as the most common microorganism, present in 40% of samples [11]. Although widely associated with DFIs, *Staphylococci* often exist as commensal organisms, particularly in the nose and throat, and the prevalence of *S. aureus* carriage is thought to be around 30% [20]. This is supported by UK data, including a community-based study which showed a *S. aureus* carriage rate of 28% [21] and a prevalence of 41% among Royal Marine recruits [22]. *Staphylococci* often colonise the skin [23, 24], and, despite following guidelines for the investigation of wound microbiology, we therefore cannot exclude the possibility that some *Staphylococcal* cultures represent skin commensals. For this reason coagulase-negative *Staphylococci* are locally not routinely reported for deep tissue swabs. Other major pathogens associated with DFIs include *Enterococci*, *Streptococci*, *Escherichia coli* and *Pseudomonas aeruginosa* [25]. However, these pathogens either occurred at a relatively low frequency or were absent from our data. It has been reported that Gram positive organisms are often more frequently isolated from DFIs in Western nations, with Gram negatives more frequently isolated from warmer climates, including Asia and Africa. The reasons for this remain unclear but could reflect a variety of environmental, clinical or personal factors [26]. The frequency of samples with no significant growth (38%) is broadly comparable with that found in similar studies in the UK [16, 19], and similar to that reported for the only other Scottish analysis of the microbiology of DFIs (45%) performed at the same hospital [11].

Given the potential breadth of associated factors and limited sample size, it was not surprising that our descriptive analysis of gender and anatomical sample site did not reveal any clear association with the broad pattern of microbiological growth observed. Although analysis of these data was limited by violation of an assumption of the Chi-Square test, the difference between the microbiological patterns reported from bone biopsies and deep tissue swabs was intriguing, but limited by the sample size. A higher frequency of polymicrobial results from bone samples could suggest that these patients had deeper and potentially more complex and chronic infections. More broadly, there are conflicting reports of the concordance of bone and tissue samples [27, 28].

Young age and male gender are among a range of factors sometimes considered to increase the carriage of *S. aureus* carriage in the community, although mixed results have been observed [20, 29]. While these factors may affect carriage rates, this study found no evidence that gender is a factor that contributes to the chance of having a *S. aureus* positive DFI. There was also no evidence to suggest that sample site or material were associated with a higher rate of *S. aureus* infection. This study therefore suggests that the rate of *S. aureus* positive DFIs may be independent of these factors, however these findings are somewhat limited by the statistical power. In general, the prevalence of commensal *S. aureus* colonisation is considered to decline with increasing age, including among hospital patients [20, 29]. However, this study found that the prevalence of *S. aureus* in DFIs was in fact associated with increasing patient age. The reasons for this are unclear, but it may reflect sample size or underlying temporal trends in the clinical state of these patients.

Antimicrobial resistance is a common finding in DFIs, with meta-analyses estimating the global prevalence of methicillin-resistant *S. aureus* in DFIs to be 15–30% [8, 9]. Previous studies in the UK have reported higher rates, ranging from 19.9 to 57.9% [16–19]. Reassuringly this analysis did not report the isolation of any MRSA and, where investigated, a high degree of sensitivity to flucloxacillin was observed. However, 27.4% resistance to clarithromycin was detected, although this is comparable with previous Scottish data [30]. The absence of notable antimicrobial resistance from these data could suggest that current hospital infection control and MRSA screening policies are effective. However, decreases in antimicrobial resistance have also previously been associated with changes in the dominance of the circulating bacterial strain [31].

This study was hindered by an inability to access more relevant clinical data. This represents a wider limitation of clinical research that encounters the use of discrete databases and systems for different clinical utilities. A further limitation is the potential for intrinsic bias because of individual patients contributing multiple results, although these most likely represented different infectious episodes from the same foot ulcer. We considered addressing this by removing duplicate results from individual patients but deemed that the greatest clinical relevance was provided by the data as presented. Provision of multiple results may therefore have also introduced a degree of bias into the antimicrobial resistance data as, for genetic or behavioural reasons, some patients may be more prone to infection with certain bacterial species. Knowledge of the microbiology of diabetic foot infections is important in helping monitor the presence of antimicrobial resistance among this at-risk population and provides evidence to guide the targeting of novel anti-infectives.

## Conclusion

In summary, this retrospective analysis has increased the understanding of the microbiology of DFIs in Scotland, provided reassuring data regarding antimicrobial resistance and suggests that the prevalence of *S. aureus* in DFIs may be associated with increasing patient age.

## Data Availability

The datasets generated during and/or analysed during the current study are available from the corresponding author on reasonable request.

## Abbreviations

DFIs: Diabetic foot infections
DFUs: Diabetic foot ulcers
DHARMa: Diagnostics for HierArchical Regression Models
MRSA: Methicillin resistant *Staphylococcus aureus*
NHS: National Health Service
QEUH: Queen Elizabeth University Hospital
UK: United Kingdom
